# Diagnosis and Treatment of Primary Tracheobronchial Tumors

**DOI:** 10.1002/cam4.70893

**Published:** 2025-04-27

**Authors:** Chen Shu, Yu‐jian Liu, Kai‐fu Zheng, Xi‐yang Tang, Meng‐chao Li, Yang Shen, Yu‐long Zhou, Wei‐guang Du, Nan Ma, Jin‐bo Zhao

**Affiliations:** ^1^ Department of Thoracic Surgery Tangdu Hospital, The Fourth Military Medical University Xi'an Shaanxi China; ^2^ Department of Cardiothoracic Surgery The 902nd Hospital of the Chinese People's Liberation Army Joint Logistic Support Force Bengbu Anhui China; ^3^ Department of Cardiothoracic Surgery Central Theater Command General Hospital of Chinese People's Liberation Army Wuhan Hubei China; ^4^ Department of General Surgery The 991st Hospital of the Chinese People's Liberation Army Joint Logistic Support Force Xiangyang Hubei China; ^5^ Department of Ophthalmology Tangdu Hospital, The Fourth Military Medical University Shaanxi China

**Keywords:** diagnosis, immunotherapy, primary tracheobronchial tumors, surgical treatment, tracheal reconstruction

## Abstract

**Background:**

Primary tracheobronchial tumors (PTBTs) are rare but life‐threatening, accounting for approximately 0.2% of all respiratory neoplasms. Owing to their nonspecific clinical symptoms, PTBTs are often initially misdiagnosed as bronchial asthma or bronchitis in the early stages. In addition, standardized treatments for PTBTs are currently lacking.

**Aims:**

This study aimed to provide a comprehensive review of this diagnostic challenge and treatment modalities of PTBTs.

**Methods:**

Drawing on the latest literature and clinical guidelines, we carried out a comprehensive and systematic analysis of PTBTs, focusing on diagnostic modalities, and evidence‐based treatment options.

**Results and Conclusions:**

Primary diagnostic methods for PTBTs include pulmonary function tests, chest radiography, computed tomography, and fiberoptic bronchoscopy. Computed tomography, and fiberoptic bronchoscopy may be the most valuable diagnostic tools for patients with PTBTs or those highly suspected of having PTBTs. Currently, there are no consensus guidelines for PTBTs, and surgery is the most effective method for treating PTBTs if the patients have indications for surgery. In addition, radiotherapy, chemotherapy and interventional therapy may be useful complementary treatments for inoperable patients. Immunotherapy may be a significant management strategy for PTBTs in the future. Further researches should concentrate on both the early identification and enhanced therapeutic management of these tumors to improve survival and diminish morbidity and mortality rates by investigating the optimal design of systematic therapy.

**Abbreviations**
3D: third dimensional
ACC: adenoid cystic carcinoma
APC: argon plasma coagulation
COPD: chronic obstructive pulmonary disease
CT: computed tomography
CXR: chest radiography
EBUS‐TBNA: endobronchial ultrasound‐guided transbronchial needle aspiration
EUS‐FNA: endoscopic ultrasound‐guided fine‐needle aspiration
FEV1: the forced expiratory volume in the first second
FEV1/FVC: the ratio of FEV1 to FVC
FiO2: fraction of inspired oxygen
FOB: fiberoptic bronchoscopy
FVC: the volume exhaled during the initial second of forced vital capacity
Gy: Gery
IT: interventional therapy
LRFS: local recurrence‐free survival
OS: overall survival
PD‐L1: programmed cell death‐ligand 1
PDT: photodynamic therapy
PFTs: pulmonary function tests
PTBTs: primary tracheobronchial tumors
QUANTEC: quantitative analysis of normal tissue effects in the clinic
RFA: radiofrequency ablation
RT: radiotherapy
SCC: squamous cell carcinoma
TBNA: transbronchial needle aspiration

## 1 Introduction

Primary tracheobronchial tumors (PTBTs) are rare but life‐threatening. The morbidity rate of PTBTs is reported to be as low as 0.01%–0.4%. These PTBTs account for approximately 0.2% of all respiratory neoplasms. The economic costs associated with tracheal, bronchial, and lung cancers were the highest, representing 15.4% among 29 types of cancers in 204 countries and territories [1, 2, 3, 4]. PTBTs can be categorized into malignant and benign tumors based on their pathological types; squamous cell carcinoma (SCC) and adenoid cystic carcinoma (ACC) are the most common pathological types, accounting for more than two‐thirds of all PTBTs [5, 6]. Other pathological types of malignant tumors include mucinous epidermoid carcinoma, carcinoids, adenocarcinoma, large‐cell carcinoma, and sarcoma. Recently, the proportion of ACC has gradually increased, surpassing that of SCC, the most frequent type of PTBTs. A recent study showed that ACC accounted for 33.7% of primary tracheobronchial malignancies, exceeding 31% of SCC [7]. The median age was significantly higher in patients with SCC than in patients with ACC, while the ratio of male to female patients was essentially similar. The pathological types of benign tumors include papilloma, smooth muscle tumors, chondroma, lipoma, mucinous adenoma, hemangioma, and nerve sheath tumors, with papilloma and smooth muscle tumors being the most common [8, 9, 10, 11]. Globally, smoking has long been recognized as the most prominent risk factor for all histological types of PTBTs among both males and females, except for ACC. In patients with ACC, the proportion of those with a smoking history is relatively low [12, 13]. Of note, for women, especially in some geographical regions, ambient particulate matter pollution, particularly fine particulate matter (PM2.5), and household air pollution from solid fuels are significant risk factors for ACC. Furthermore, alcohol consumption [14], unhealthy dietary habits [15], physical inactivity [16], hypertension [17], lipid disorders, and occupational exposure to certain chemicals have been found as risk factors of PTBTs [18, 19, 20].

PTBTs, both benign and malignant pathological types, are mainly characterized by luminal narrowing, leading to ventilation obstruction. Luminal narrowing usually manifests as dyspnea, shortness of breath, wheezing, cough, and hemoptysis [21, 22, 23, 24]. Owing to their nonspecific clinical symptoms, PTBTs are often misdiagnosed as bronchial asthma or bronchitis in the early stages, especially in the case of ACC. The tumor is usually quite large at the time of diagnosis and may even invade the surrounding tissue. Nevertheless, due to the tardy diagnosis of PTBTs and the hardship in distinguishing them from chronic obstructive pulmonary disease (COPD) and other common respiratory‐related chronic diseases, the likelihood of surgery in patients was significantly decreased [25]. Therefore, PTBTs should be considered for patients who have been repeatedly treated with conventional treatments with no effect, and relevant examinations should be performed. The survival probabilities of patients vary significantly among individuals with different pathological types of PTBTs. According to the Surveillance, Epidemiology, and End Results database, patients with ACC have a higher 5‐year cause‐specific survival rate than those with neither SCC nor ACC histology, as well as those with SCC histology [26, 27].

To date, no standard treatment for PTBTs exists, and the predominant treatment approach is surgical intervention. Currently, the primary treatment modalities for patients who cannot undergo surgery include radiotherapy, chemotherapy, interventional therapy, and immunotherapy [28, 29, 30]. Therefore, it is clinically valuable to continue exploring new treatment options for patients with PTBTs. Here, we mainly discuss the diagnosis and treatment of PTBTs to provide an overview of this disease and offer guidelines for clinicians to better identify and manage PTBTs.

## 2 Diagnosis

**FIGURE 1:**
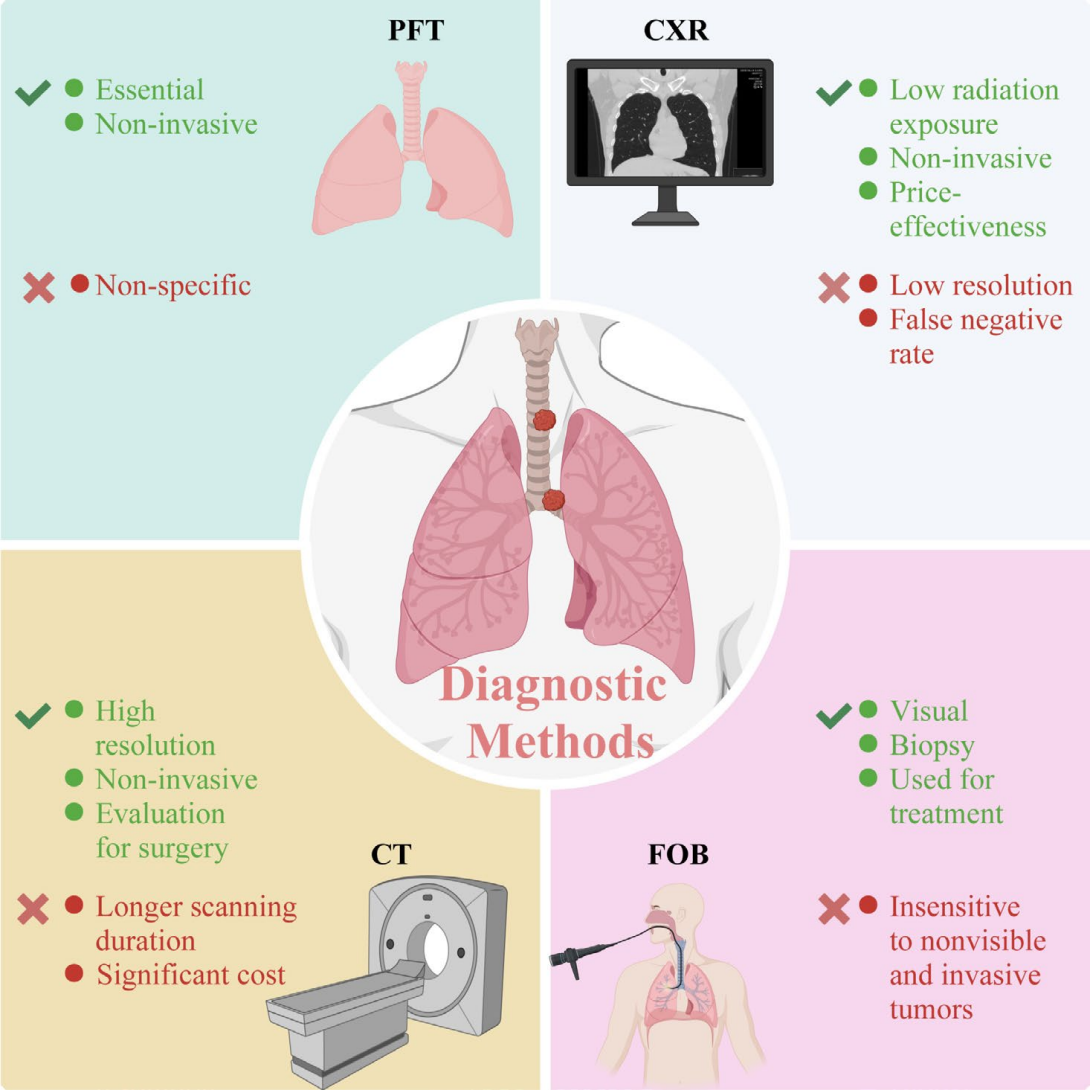
Overview of diagnostic methods for PTBTs. Diagnostic methods for PTBTs mainly include pulmonary function tests (PFTs), chest radiography (CXR), computed tomography (CT), and fiberoptic bronchoscopy (FOB).

Diagnostic methods for PTBTs mainly include pulmonary function tests (PFTs), chest radiography (CXR), computed tomography (CT), and fiberoptic bronchoscopy (FOB) (Figure 1). Among all listed diagnostic methods for PTBTs, PFTs and CXR may function as a screening tools. In addition, CT can be utilized to diagnose PTBTs, and FOB is recognized as the gold standard for diagnosing PTBTs. A summary of diagnostic methods, including advantages, shortcomings, and diagnostic value, is provided in Table 1.

**TABLE 1:** Summary of diagnostic methods for PTBTs.

Diagnostic methods	Advantages	Shortcomings	Diagnostic value
Pulmonary function tests (PFTs)	Essential Noninvasive	Non‐specific	Screening tool
Chest radiography examination (CXR)	Low radiation exposure Noninvasive Price‐effectiveness	Low resolution False negative rate	Screening tool
Computed tomography (CT)	High resolution Noninvasive Evaluation for surgery	Longer scanning duration Significant cost	Diagnostic tool
Fiberoptic bronchoscopy (FOB)	Visual Biopsy Used for treatment	Insensitive to nonvisible and invasive tumors	Gold standard

Abbreviations: CT, computed tomography; CXR, chest radiography; FOB, fiberoptic bronchoscopy; PFTs, pulmonary function tests.

### 2.1 Pulmonary Function Tests

Pulmonary function tests (PFTs) are essential, non‐invasive diagnostic tools for some respiratory diseases, which are usually applicable to moderate to large‐size tumors, and are especially critical for the diagnosis, care, and prognosis of chronic respiratory disorders [31, 32]. However, it is not advisable to perform PFTs in patients with severe cardiopulmonary impairment, inability to comply with the operator's instructions, or other unstable conditions [33]. For patients with PTBTs, the FEV1/FVC ratio and peak inspiratory and expiratory flow rates decreased when the tracheal lumen narrowed. Orton et al. reported a case in which a patient who underwent PFT exhibited a notch in the flow‐volume curve, which was further diagnosed as PTBTs [34]. Therefore, the flow‐volume curve can be utilized to assess tracheal obstruction. Incidentally, 50% of the patients' forced spirometry changes depending on the cause, degree, location, and type of tracheal obstruction [35]. Therefore, flow‐volume curves can be employed as screening tools for PTBTs. PTBTs should be suspected when the flow‐volume curve demonstrates flattening of the expiratory limb [36, 37]. However, since the flow‐volume curve is not a specific diagnostic tool for PTBTs and may be complicated by concurrent respiratory diseases such as asthma, COPD, etc., it is important to consider further examinations using other screening tools when PTBTs are clinically suspected.

### 2.2 Chest Radiography Examination

Chest radiography (CXR) is the most common imaging test in clinical practice and is an important tool for identifying many types of chest tumors. The major benefits of CXR include low radiation exposure, cost‐effectiveness, and appropriate sensitivity to a wide range of disorders [38, 39, 40]. However, owing to the anatomical location of the trachea, the interpretation of CXR results of PTBTs is susceptible to the influence of the imaging of surrounding tissues, including the mediastinum, heart, and sternum. Consequently, most PTBTs, especially those in the early stages, have negative CXR results, and only 18%–28% of patients with PTBTs are appropriately diagnosed using CXR [41]. Although CXR has limited value as a confirmatory test for PTBTs, it is efficient as an initial diagnostic tool and method to exclude other histological diseases. Owing to the rapid advancement in deep learning and artificial intelligence technologies, CXR may play a vital role in the diagnosis of PTBTs in the future [42, 43].

### 2.3 Computed Tomography

Compared with CXR, computed tomography (CT) has a higher resolution. It can provide a clearer demonstration of the location, extent of infiltration, invasion, surrounding lymph nodes, and mediastinal metastases of PTBTs, which is of great significance for the diagnosis and surgical treatment of PTBTs [44, 45]. However, CT may not be advisable as an initial screening imaging test because of its higher cost and longer scanning duration (range, 15–30 min), compared with CXR [46]. Meanwhile, CT is not recommended for patients with severe heart failure, an inability to comply with the operator's instructions, or other unstable conditions. Nevertheless, some studies have shown that traditional CT techniques are not sufficiently accurate in the evaluation of PTBTs, with the percentage of false negatives being up to 9% [47]. Approximately 9% of patients evaluated by CT and deemed fit for surgical resection are found to have tumor invasion exceeding the scope of CT evaluation, rendering intraoperative intervention unfeasible [48, 49]. In recent years, the diagnostic value of CT for PTBT has increased with the development of CT imaging technology and image processing software. For example, multidetector CT, third dimensional (3D) reconstruction, and virtual simulation endoscopy have enabled more accurate assessment of PTBTs.

### 2.4 Fiberoptic Bronchoscopy

Fiberoptic bronchoscopy (FOB) is recognized as the gold standard for diagnosing PTBTs, enabling visual observation of the size, morphology, and location of tumors, and the degree of tracheal lumen obstruction [50, 51]. The indications for FOB can be classified into two categories: Diagnostic and therapeutic. FOB can be used for patients with atelectasis, hemoptysis, localized wheezing, and chest radiography findings suggestive of neoplasms. For therapeutic indications, FOB is primarily used for conditions such as hemoptysis, acute lobar collapse, challenging endotracheal intubation, and endoscopic tumors [52]. However, FOB may not be advisable for patients with conditions such as severe cardiopulmonary disease, hemodynamic instability, serious hypoxemia, and coagulation disorders [53, 54]. A major advantage of FOB is that a biopsy can be performed simultaneously with the examination to obtain a pathological diagnosis and perform treatment. Nonetheless, Lam et al. concluded that FOB did not possess satisfactory sensitivity for nonvisible tumors (approximately 50%) [55]. The development of bronchial ultrasound technology and electromagnetic navigation may further compensate for the deficiency of FOB in assessing the degree of PTBTs [56, 57, 58]. When it becomes essential to assess whether the tumor has invaded the lymph nodes, direct sampling represents the most definitive methodology for evaluating lymph node involvement. Minimally invasive procedures employ needle biopsy techniques to procure tissue specimens from mediastinal lymph nodes. These needle biopsy modalities encompass transbronchial needle aspiration (TBNA), transthoracic needle aspiration, endoscopic ultrasound‐guided fine‐needle aspiration (EUS‐FNA), and endobronchial ultrasound‐guided transbronchial needle aspiration (EBUS‐TBNA) [59, 60, 61]. EBUS‐TBNA, a minimally invasive method for mediastinal biopsy under direct real‐time endobronchial ultrasound guidance, can access the paratracheal lymph node stations (groups 2R, 2 L, 4R, 4 L), subcarinal lymph nodes (group 7), and hilar, interlobar, and lobar lymph nodes (groups 10, 11, and 12). Furthermore, mediastinoscopy facilitates the direct interrogation of specimens derived from the paratracheal lymph nodes (lymph node stations 2R, 2 L, 4R, 4 L), the pre‐vascular subcarinal lymph nodes (lymph node station 7), and the hilar lymph nodes (lymph node station 10) [62]. However, as patients with PTBTs have varying levels of tracheal stenosis, placing fiberoptic bronchoscopes in the trachea for examination may further aggravate the patient's hypoxic symptoms, leading to asphyxia or even death in critical cases [63]. Therefore, conducting a thorough assessment before the examination and preparing for emergency measures is important. For patients with tracheal tumors that severely obstruct the trachea (< 50% lumen occlusion), CT rather than FOB examinations should be performed to avoid grave consequences [64].

## 3 Treatment

**FIGURE 2:**
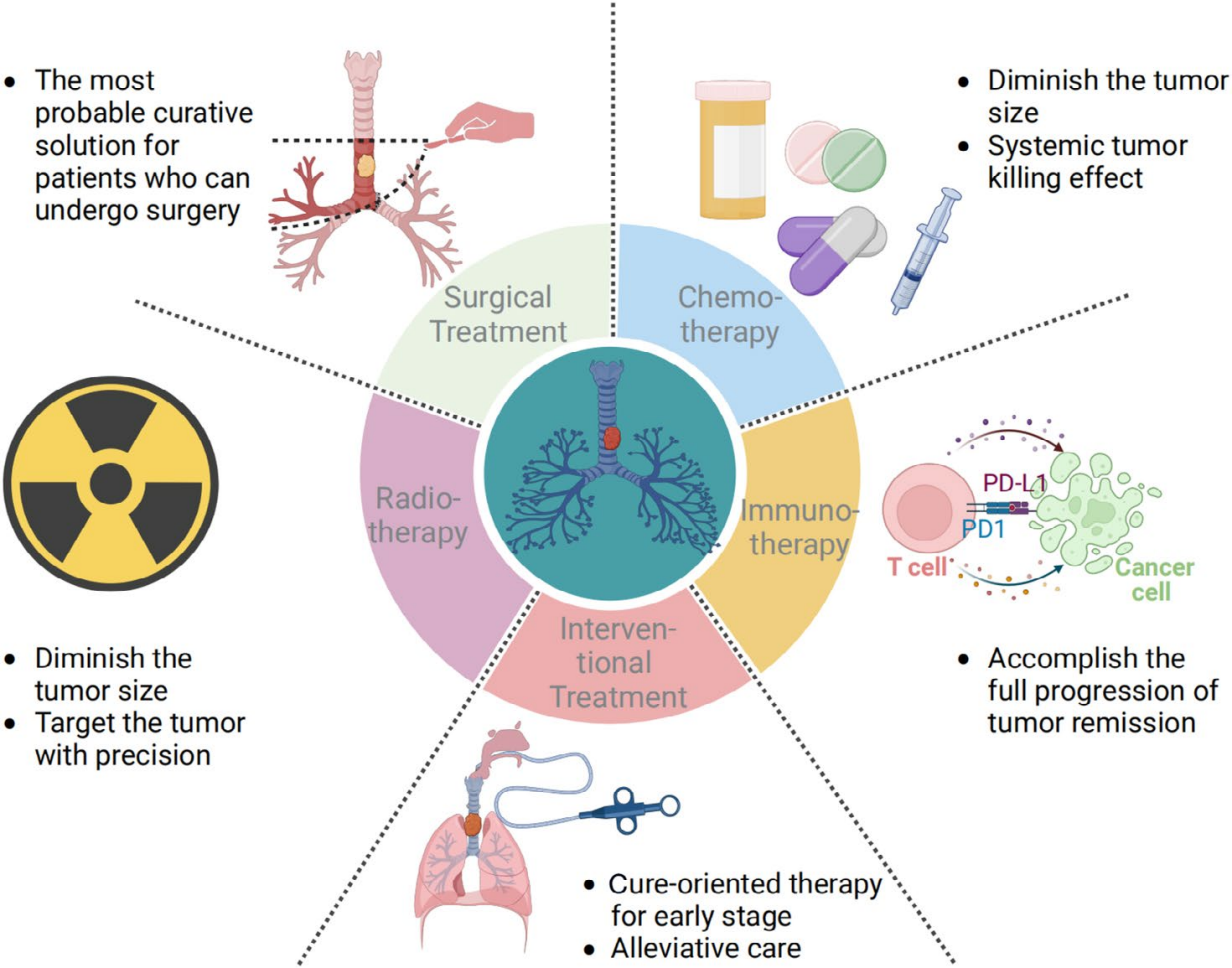
Current major treatment modalities for PTBTs. Treatment approaches for PTBTs primarily contain surgery, chemotherapy, radiotherapy, interventional treatment, and immunotherapy. Surgical treatment is the most probable curative solution for PTBTs.

In PTBTs, patients whose tumor size is less than 5–6 cm (half the length of the trachea) find surgical treatment to be the most effective approach. Currently, the conservative treatment methods for PTBTs include radiotherapy, chemotherapy, and interventional therapy. In recent years, some case reports have shown that PTBTs respond well to immunotherapy [65, 66, 67]; thus, immunotherapy could be an important means of treating PTBTs in the future (Figure 2). A summary of each treatment approach listed above can be seen in Table 2.

**TABLE 2:** Summary of treatment approaches for PTBTs.

Treatment		Advantages	Shortcomings	Indications	Contraindications
Surgical treatment	Tracheal sleeve Resection with end‐to‐end anastomosis	Primary surgical means	Anastomotic tension issues	Tumor size < 6 cm	Lymph node invasion > 50% tracheal involvement Prior RT > 60 Gy
	Autologous tissues	No immunosuppression Preserve blood supply	Technical difficulty Obstruction of cilium	Resection > 6 cm (adults) or > 30% (children)	Intolerance to reconstruction
	Allograft trachea	Preserve native structure Achieve revascularization	Immunosuppressive response Donor deficiency		Malignant tracheal neoplasms
	Allograft aorta transplantation	Low immunogenicity Extensive sources	Stents placement Cilia‐free swing function		Same as Autologous tissues
	Tissue‐engineered trachea	No immunosuppression Realize regeneration	Difficulty with revascularization, epithelialization and chondrogenesis		Same as Autologous tissues
Chemotherapy	—	Diminish tumor size Systemic tumor killing	Adverse drug reaction	Pre/postoperative adjuvant Metastatic disease	Chemotherapy intolerance
Immunotherapy	—	Full tumor remission	Unknown clinical efficacy	Immune checkpoint‐sensitive tumors	Immunotherapy intolerance
RT	—	Diminish tumor size Precise tumor targeting	RT‐related complications	Pre/postoperative adjuvant Palliative therapy	Severe respiratory Cardiac dysfunction
IT	Electrocautery	Simple and cost‐effective	Incomplete tissue debulking	Early‐stage central airway tumors	FiO2 > 40%
	Cryotherapy	Modulate wound healing Mitigate fibrosis	Delayed effect and Complex	Benign or malignant airway obstruction	Bronchoscopy intolerance Instable airway
	Laser therapy	Treat deep‐rooted organizations	Perforation and bleeding risk	Intraluminal neoplasms Obstructive lesions of the airway	External pressure FiO2 > 40% Hypoxemia
	PDT	Less bleeding	Delayed effect	Same as Laser therapy	Lesions adjacent to the carina
	APC	Easy and cheap Immediate hemostasis	Incomplete tissue debulking Gas embolism risk	Malignant obstruction Main bronchus lesions	Same as Electrocautery
	Brachytherapy	Feasible Precise	Delayed effect	Malignant obstruction Palliative care	Surgical patients
	RFA	Valid and microinvasive	Thermal injury risk	Central malignant obstruction Benign tumors	Same as Electrocautery
	Airway stents	Safety and rapid symptom relief	Granulation tissue formation	Malignant compression Fistulas and Stenosis	External vascular compression

Abbreviations: APC, Argon plasma coagulation; FiO2, Fraction of inspired oxygen; Gy, Gery; IT, Interventional treatment; PDT, Photodynamic therapy; RFA, Radiofrequency ablation; RT, Radiotherapy.

### 3.1 Surgical Treatment

#### 3.1.1 Tracheal Sleeve Resection With End‐To‐End Anastomosis

Surgical treatment is the most probable curative solution for PTBTs. Tracheal sleeve resection with end‐to‐end anastomosis is well accepted and possibly the optimal surgical method for PTBTs [68]. Owing to the special characteristics of the tracheal anatomy, the length of tracheal resection is limited. The above factor also accounts for the low surgical resection rate of PTBTs; some studies have shown that 67% of patients cannot be treated surgically because of the tracheal resection length. Meanwhile, the patient's basic condition, including a history of chest radiation, advanced age, and kyphosis, may seriously affect the success rate of surgery. For patients with a history of chest radiotherapy, especially when a dose of over 3500 cGy was conducted 3 weeks before the resection [30, 69], tracheal sleeve resection with end‐to‐end anastomosis should be performed with caution, as this may compromise the tracheal blood supply and increase the risk of postoperative tracheostomy fistula [70]. While it is generally accepted that resection‐anastomosis is feasible for lesions extending to less than half of the tracheal length (5–6 cm), this theoretical limit can be significantly lowered due to certain characteristics listed above or in cases of carinal invasion. Furthermore, surgical intervention is not indicated in cases of proximity to mediastinal and lymph node invasion, involvement of more than 50% of the trachea, and distant metastases. During surgery, the following potential problems should be noted: First, the incision approach is an essential part of the successful resection of PTBTs, and improper selection of the incision route can lead to insufficient exposure of the tumor location, which can further complicate the resection. For upper airway tumors, a cervical collar incision is recommended for improved visualization and surgical success, whereas for middle tracheal tumors, a central incision is more appropriate for resection. For tumors in the inferior trachea and/or carina, surgery can be performed through a posterior lateral incision or median sternal split [71, 72]. Second, the free length of the trachea should not be too long in order to avoid damaging too many blood vessels, which can affect the healing of anastomosis and lead to tracheal anastomotic fistula [73]. Third, the trachea should be dissected carefully, with particular attention paid to the recurrent laryngeal nerve, as it is an essential structure prone to damage during tracheal surgery. Damage to the recurrent laryngeal nerve can significantly affect the patient's postoperative quality of life. Finally, the most crucial principle of tracheal tumor resection is to achieve tension‐free anastomosis, which is a key factor in ensuring surgical success. Studies have shown that complete resection of tracheal tumors requires a minimum surgical margin of 0.5 cm from the tumor. Therefore, tissues at the margin should undergo intraoperative freezing to ensure thorough tumor removal. However, owing to the limitation of the length of tracheal resection, achieving this 0.5 cm surgical margin may be difficult. Thus, tension‐free anastomosis of the trachea should be ensured, and tracheal anastomosis should not be overly tense to achieve radical resection of the tumor. Currently, the main approach is to mobilize the peritracheal tissues and structures to achieve tension‐free anastomosis, including the laryngeal suprahyoid region, pericardium, pulmonary hilum, and inferior pulmonary ligament [74]. Once resection is successfully performed, various factors influencing the prognosis of PTBTs should be evaluated. Liu et al. identified age and tumor size as prognostic factors after analyzing 72 patients who underwent surgery for PTBTs from 2004 to 2020 [75]. In addition, Ahn et al. identified clinical stage as a significant prognostic factor [21]. Similarly, He et al. reported that age, tumor size, advanced extension, and surgery could significantly impact the prognosis of PTBTs, drawing from 40 years of experience [76]. Hence, early diagnosis and prompt surgical treatment may play vital roles in the prognosis of PTBTs.

#### 3.1.2 Tracheal Reconstruction

It is commonly believed that when the length of tracheal resection exceeds 50% in adults or 30% in children, tension‐free anastomosis will fail, regardless of whether the adjacent tissue has been loosened [77]. This theoretical limit can vary with factors such as age, anatomical variations, and previous treatments. Therefore, tracheal substitutes may serve as appropriate supplements. Tracheal substitutes mainly include autologous tissues, allograft trachea, allograft aorta, artificial trachea, and tissue‐engineered trachea [77, 78, 79, 80]. Autologous tissue is the most widely used alternative to tracheal tissue. Autologous tissues reported in recent studies include the radial forearm free flap [81], pectoralis major muscle flap [82], sternocleidomastoid muscle flap, intercostal muscle flap, greater omental flap [83], and anterolateral thigh free flap [84]. Although this technique has certain shortcomings, including rib cage calcification and complex procedures—which limit its application in elderly patients with advanced age, diaphragmatic insufficiency, or limited coughing function—it preliminarily confirms the feasibility of autologous tissue‐constructed tracheal substitutes for the surgical treatment of PTBTs. Therefore, further comprehensive research should be conducted in subsequent studies to overcome the existing drawbacks of autologous tissues and to provide more surgical opportunities for patients with long‐segment PTBTs.

The allograft trachea has limited application value for the surgical treatment of PTBTs due to the associated immunosuppressive response. Patients require persistent use of immunosuppressive drugs to prevent graft‐versus‐host reactions, which may promote the progression and recurrence of PTBTs [85]. Delaere et al. successfully performed allogeneic tracheal transplantation in a patient with recurrent tracheal chondrosarcoma using an allogeneic trachea pre‐vascularized for 3 months. The patient did not exhibit tumor progression while using immunosuppressants; this may be because tracheal chondrosarcomas are inherently low‐grade malignant tumors with a slow progression rate [85]. Therefore, allogeneic tracheal transplantation is not recommended for airway reconstruction in malignant tracheal neoplasms such as SCC, ACC, and mucoepidermoid carcinoma.

Unlike allogeneic trachea grafts, allogeneic aortic grafts do not require immunosuppressive therapy, thus promoting their use as tracheal substitutes for long‐segment PTBTs. In 2018, a clinical trial involving airway transplantation with the utilization of stented aortic matrices confirmed the feasibility for complex tracheal and bronchial reconstruction [79]. In addition, Martinod et al. reported the preliminary results of 13 patients with PTBTs who underwent airway reconstruction with a stented aortic matrix, of which 11 were ACC, 1 was SCC, and 1 was a mucoepidermoid carcinoma case. After a follow‐up of up to 3 years and 7 months, the results indicated that 61.5% (8/13) of the patients suffered from post‐transplantation complications, which principally comprised tracheal granulomas and pneumonia. The overall survival (OS) rate was 84.6% (as reported in the preliminary results of tracheal replacement using stented aortic matrices for primary extensive tracheal cancer) [86]. Notably, in a cohort of 35 patients over a 25‐year period, the TRITON‐01 study showed rather low 30‐day postoperative mortality and morbidity rates and a relatively low incidence of long‐term complications. This suggests that the use of stented aortic matrices for airway replacement may be recommended as a regular treatment option [87]. Therefore, allogeneic aortic graft transplantation is a promising method for treating long‐segment tracheal defects in PTBTs. However, there are some limitations to allogeneic aortic graft transplantation, such as the shortage of sources, the high price of the aorta, and the removal time of stents. Further research is needed, and more scientists should work together to solve these problems.

Theoretically, a tissue‐engineered trachea may be the ideal substitute for long‐segment tracheal defects after the surgery for PTBTs as no immunosuppressive therapy is involved during the course of the treatment. The main challenges of a tissue‐engineered trachea lie in revascularization, epithelialization, and chondrogenesis of the substitute. Although numerous encouraging achievements have been made through the efforts of many scientific researchers [88], more experimental trials of tissue‐engineered trachea technology are required in the field of regenerative medicine before clinical applications can be realized.

### 3.2 Chemotherapy

The effects of chemotherapy on PTBTs remain unclear due to the limited availability of case reports. The main chemotherapeutic agents for tracheal tumors utilized in current research include platinum, etoposide, 5‐fluorouracil, and paclitaxel, which may cause side effects, including diarrhea, dermatitis, esophagitis, and peripheral neuropathy [89, 90]. Chemotherapy mainly functions as an adjuvant treatment to radiotherapy or immunotherapy for patients with unresectable PTBTs. Jiang et al. reported two cases that adopted chemotherapy plus immune checkpoint inhibitors as neoadjuvant treatments and achieved satisfactory outcomes. One patient underwent three cycles of chemotherapy (docetaxel 75 mg/m2 + carboplatin) plus nivolumab (360 mg) every 21 days and was disease‐free for 18 months after surgery due to shrinkage of the cancerous tumors. Another patient underwent three cycles of chemotherapy (paclitaxel 200 mg/m2 + carboplatin) plus nivolumab (360 mg) every 21 days and eventually had the opportunity to have surgery [66]. These two cases revealed satisfactory clinical outcomes of chemotherapy plus immunotherapy for PTBTs, which may diminish the need for resection. Despite the satisfactory results of the case reports, further prospective investigations are warranted to evaluate the effectiveness of chemotherapy for PTBTs.

### 3.3 Immunotherapy

Immunotherapy is a novel therapeutic approach that targets immune checkpoint molecules, which has shown promising therapeutic outcomes in multiple types of neoplasms. However, its applicability to PTBTs remains uncertain. In contrast to other palliative therapies such as chemotherapy, immunotherapy can accomplish the full progression of tumor remission. Current studies on immunotherapy for PTBTs are limited to case reports. Massachusetts General Hospital first published a study in 2017 on a 67‐year‐old man with recurrent tracheal SCC who received 7 months of navulizumab treatment, with the aim of achieving complete remission. Despite multiple therapies and tumor reduction surgeries after recurrence, he ultimately died from recurrent tumor growth blocking the lumen [67]. In 2019, a research team used pembrolizumab to treat patients with recurrent tracheal SCC. In 2019, Maller et al. used pembrolizumab to treat patients with recurrent tracheal SCC and observed complete tumor remission 3 months after therapy [91]. Although the results indicate the potential for long‐term remission in PTBTs through immunotherapy [67, 91], they were not convincing because of the small sample size, ambiguous theoretical mechanism, and inadequate representation. Understanding the tumor immune microenvironment is fundamental to immunotherapy. The basic principle involves activating CD8+ T cells and other tumor‐killing immune cells, a prerequisite for the clinical immunotherapy implementation [92, 93]. To date, only a few studies have investigated the immune microenvironment of PTBTs. Wang et al. analyzed 25 cases of ACC and conducted whole‐exome sequencing and T‐cell receptor sequencing and found no mutations in common driver genes of lung cancer (such as NOTCH1) in ACC. They also found that the mutation load of ACC was 3.67, which was far lower than that of other solid tumors. Most importantly, most ACC cases were negative for programmed cell death‐ligand 1 (PD‐L1), with low CD3+ and CD8+ T cell infiltration. This led to the conclusion that immunotherapy, which is applicable to NSCLC, may not necessarily be suitable for ACC [94]. Zheng et al. analyzed 16 cases of ACC and five cases of SCC, assessing the expression of PD‐L1 and the infiltration of CD8+, CD16+, CD68+, CD163+, and FOXP3+ cells using immunohistochemistry. SCC samples exhibited higher PD‐L1 expression and FOXP3+ cell infiltration, compared with ACC samples, even surpassing CD8+, CD16+, and CD163+ levels. In contrast, FOXP3+ cell infiltration was lower than that of CD8+, CD16+, and CD68+ cells [95].

Despite the lack of studies on the immune microenvironment of tracheal tumors, the conclusions of the above studies are basically consistent: Compared with ACC of the trachea, SCC may benefit more from immunotherapy due to higher levels of PD‐L1 expression and a higher degree of immune cell infiltration. Therefore, further research is essential to investigate the role of immunotherapy in PTBTs, including potential markers of immunotherapy, neoadjuvant immunotherapy, and postoperative immunotherapy protocols. A larger number of studies should be conducted to explore the immune microenvironment of PTBTs in greater depth and expand the application of immunotherapy in PTBTs.

### 3.4 Radiotherapy

Radiotherapy (RT) for PTBTs is usually performed as an adjuvant therapy after surgery, especially for cases involving positive margins. It also served as a palliative therapy for inoperable patients aimed at alleviating symptoms associated with disorders, reducing pain, and relieving airway obstruction [96]. Several studies have suggested that RT can increase the OS rates for PTBTs and decrease the likelihood of tumor recurrence, metastasis, and cancer‐related mortality [97, 98]. Ran et al. summarized 1252 cases of ACC and demonstrated that 36.4% of patients who received postoperative RT had 5‐year survival rates of up to 97.3%, higher than that of patients who underwent surgery alone (86.4%) [98]. Therefore, surgery with postoperative RT is considered the most effective therapeutic option for PTBTs [24], with ACC being more sensitive to RT than SCC [99]. In a study of 18 patients with ACC who received 66–72.6 Gy RT, side effects of esophagitis, pneumonitis, tracheal stenosis, hoarseness, radiation‐induced lung injury, and hematological toxicities were observed, and the 2‐year OS and progression‐free survival rates were 100% and 61.4%, respectively [100]. Dierkesmann et al. reported that RT may cause large defects in the wall of the trachea [101]. Kaminski et al. suggested that RT can influence the prognosis of tracheal anastomosis. Therefore, it is generally recommended to start RT at approximately 2 months postoperatively [28]. Patients with severe respiratory insufficiency, grossly impaired heart function, a previous diagnosis of non‐lung cancer, or all the listed criteria were not eligible for RT [102].

For patients who received adjuvant RT or definitive RT, the RT dose may be a significant influencing factor [103, 104]. After analyzing the results of 20 patients who received adjuvant and definitive RT, Je et al. concluded that patients treated with higher doses of brachytherapy‐intensive RT had a better 5‐year OS of 83.3%, and that most side effects were tolerable [103]. Dracham et al. reported that improved prognosis was reported more frequently in patients who received high doses of RT (≥ 66 Gy) and had a 5‐year local recurrence‐free survival (LRFS) rate of 75% versus 16.7% [104]. According to QUANTEC (quantitative analysis of normal tissue effects in the clinic) and some other recommendations, the recommended radiation dose for ACC should be less than 80Gy and greater than 70Gy, while for SCC, it should exceed 60Gy. Following these guidelines can help to reduce the late radiotoxicity associated with central airway stenosis risk [105]. With the advancement of precision radiation technology, such as image‐guided RT, stereotactic RT, and volume‐rotation intensity‐modulated RT, RT can be delivered to improve tumor control rate without additional side effects [106, 107]. However, some studies have suggested that RT may not confer a survival benefit in patients who received adjuvant RT or definitive RT [27, 99, 108]. After investigating a total of 300 patients who received adjuvant RT, Yusuf et al. concluded that adjuvant RT had no statistically significant effect on OS (*p* > 0.05) in patients with PTBTs who underwent surgery [99]. Similarly, Yang et al. reported a similar 5‐year survival rate in patients who did and did not receive adjuvant RT (82% vs. 82.4%; *p* = 0.80) [108]. Although these studies negated the significance of RT in the treatment of PTBTs, there were some confounding factors, such as the fact that patients who underwent postoperative RT had more severe diseases than those who were eligible for surgical resection. Therefore, a more rigorous examination of the therapeutic properties of RT is required.

### 3.5 Interventional Therapy

Interventional therapy (IT) can be beneficial for both cure‐oriented therapy of early‐stage PTBTs, especially those located in the central airways, and alleviative care. For individuals with early‐stage PTBTs, endoscopic resection of the tumors may even result in a cure; however, it often appears to yield positive margins and requires postoperative RT [109]. In inoperable individuals, IT is an essential means of relieving respiratory obstruction from PTBTs and decreasing the tumor volume to achieve airway recirculation. IT includes electrocautery, cryotherapy, laser therapy, photodynamic therapy (PDT), brachytherapy, radiofrequency ablation (RFA), argon plasma coagulation (APC), and airway stents [110, 111, 112, 113]. The well‐adopted IT tactics are as follows: (1) coagulation of tumors to minimize bulk bleeding, (2) debulking, and (3) placement of extra stents if significant luminal narrowing remains [110]. Electrocautery, laser therapy, APC, and airway stenting can offer instant alleviation, whereas cryotherapy, PDT, and brachytherapy have delayed effects. Boxem et al. demonstrated that electrocautery and APC are cheaper, easier to operate, and simpler to apply in clinics than other ITs, such as laser therapy [114]. They can achieve immediate tumor hemostasis and remove obstructing tumors through the heating effect. However, owing to the superficial and heating effects, electrocautery and APC are mainly indicated for carcinomas in situ and superficial neoplasms located in the central airway. Electrocautery can also be used in the lobar and segmental bronchi, whereas APC is typically used to treat bronchial sections starting from the acute angle of the main airway because of their unique characteristics. Furthermore, neither electrocautery nor APC is recommended for use in individuals who require plenty of oxygen (fraction of inspired oxygen (FiO2) > 40%) [115, 116, 117]. Cryotherapy is an efficient and reliable treatment for both benign and malignant airway obstructions. It regulates wound healing and mitigates fibrosis [118]. Owing to its good heat deposition function used to treat deep‐rooted organs, laser therapy is widely utilized for treating intraluminal neoplasms and obstructive lesions of the airway. The obvious limitations of laser therapy are its inability to significantly enhance respiratory function and the risk of airway perforation bleeding [115]. Bolliger et al. summarized that contraindications for laser therapy include external pressure, hypoxemia, and coagulation disorder [110]. With the experiences from 500 patients treated with PDT, Ross et al. revealed that PDT was suitable for early‐stage airway tumor lesions, associated with tolerable complications, and easy to deal with [111]. Zhang et al. evaluated 23 patients with PTBTs treated with PDT and their clinical responses and recurrence‐free survival. The results suggested that the mean recurrence‐free survival of 23 patients with PTBTs was 8.9 ± 1.8 months (range, 1.3–22.0 months). Approximately 21 patients showed significantly ameliorated clinical conditions and experienced endurable complications [119]. The indications for PDT are the same as those for laser therapy, with a relative contraindication being lesions adjacent to the carina [120]. Brachytherapy can offer a feasible and precise measure for alleviating malignant neoplasms associated with airway obstruction or providing palliative care for intraluminal neoplasms, which can have a rapid impact [121]. Proper placement of brachytherapy catheters may be the most significant issue that requires attention, as it influences the evaluation of therapeutic effects and complications (e.g., radioactive esophagitis) [122]. Jie et al. reported that brachytherapy was the preferred treatment modality for tracheal lobular capillary hemangioma and concluded that brachytherapy performed well in cases with profound and massive defects [123]. RFA is a valid and microinvasive procedure for both malignant obstruction of the central airway and benign tracheal tumors. Liu et al. utilized RFA to treat six patients with tracheal pleomorphic adenoma with a 5‐year recurrence‐free rate [124]. However, RFA may be limited by thermal damage to the adjacent tissue and the potential for tracheal injury. RFA is considered to have the same contraindications as electrocautery. Airway stents can be used in conjunction with the other ITs mentioned above. Airway stents are primarily composed of silicone, metal, or a combination of both materials. Silicone stents are characterized by ease of placement and retrievability, enabling sequential deployment when necessary. However, their insertion typically requires general anesthesia and may be associated with higher migration rates compared to metallic alternatives. Conversely, metal stents offer enhanced bronchoscopic maneuverability, superior resistance to migration, and improved tolerance to extrinsic compression. Despite these advantages, their permanent nature renders them unsuitable for scenarios requiring stent removal, thereby contraindicating their use in benign airway diseases. As such, metallic stents are generally reserved for palliative management of malignant obstruction [125, 126]. The indications for airway stents are as follows: (1) alleviating extraneous compression from neoplasms or lymph nodes, (2) stabilizing airway patency after endoscopic resection of intraluminal carcinomas, (3) blocking malignant fistulas, such as stumpy fissures or tracheoesophageal fistulas, and (4) addressing benign narrowing. External vascular pressurization of the airway is regarded as an absolute contraindication for airway stents [127, 128]. Considering the complications of stents, such as dislocation, infection, and granulation, it is essential to conduct a rigorous evaluation when determining the appropriate placement of an endotracheal stent to maximize the benefit to the patient.

## 4 Summary

PTBTs remain an uncharted territory for many thoracic surgeons due to their low morbidity rates. CT and FOB may be the most valuable diagnostic tools for patients with PTBTs or those highly suspected of having PTBTs. Currently, there are no consensus guidelines for PTBTs, and surgery is the most effective method for treating PTBTs if the patients have indications for surgery. An effective solution for tracheobronchial replacement, such as the use of stented aortic matrices, could increase the number of patients operated on and, therefore, OS. In addition, chemotherapy, RT, and IT may be useful complementary treatments for inoperable patients. Immunotherapy may be a significant management strategy for PTBTs in the future. Further research should concentrate on both the early identification and enhanced therapeutic management of these tumors to improve survival and diminish morbidity and mortality rates by investigating the optimal design of systematic therapy.

## Author Contributions

**Chen Shu:** writing – review and editing, writing – original draft, conceptualization, investigation, methodology, validation, resources, visualization. **Yu‐jian Liu:** writing – review and editing, writing – original draft, visualization. **Kai‐fu Zheng:** writing – review and editing, writing – original draft. **Xi‐yang Tang:** software, formal analysis, writing – original draft. **Meng‐chao Li:** investigation, supervision, writing – original draft. **Yang Shen:** investigation, validation, writing – original draft. **Yu‐long Zhou:** data curation, supervision, writing – original draft. **Wei‐guang Du:** investigation, validation, writing – review and editing, writing – original draft. **Nan Ma:** visualization, conceptualization, project administration, writing – review and editing. **Jin‐bo Zhao:** visualization, validation, investigation, funding acquisition, project administration, formal analysis, resources, writing – review and editing.

## Conflicts of Interest

The authors declare no conflicts of interest.

## Data Availability

The authors declare that data and supplementary files of this study are open access. All data generated or analyzed during this study are included in this published article.
